# Serum Biomarkers of Endothelial Dysfunction in Fabry Associated Cardiomyopathy

**DOI:** 10.3389/fcvm.2018.00108

**Published:** 2018-08-15

**Authors:** Jefferson Loso, Natalie Lund, Maxim Avanesov, Nicole Muschol, Susanne Lezius, Kathrin Cordts, Edzard Schwedhelm, Monica Patten

**Affiliations:** ^1^Department of General and Interventional Cardiology, University Heart Center Hamburg, Hamburg, Germany; ^2^Department of Diagnostic and Interventional Radiology, University Medical Center Hamburg- Eppendorf, Hamburg, Germany; ^3^Department of Pediatrics, University Medical Center Hamburg- Eppendorf, Hamburg, Germany; ^4^Department of Medical Biometry and Epidemiology, University Medical Center Hamburg- Eppendorf, Hamburg, Germany; ^5^Department of Experimental Pharmacology and Toxicology, University Medical Center Hamburg- Eppendorf, Hamburg, Germany; ^6^DZHK (German Center for Cardiovascular Research e.V.), Hamburg, Germany

**Keywords:** matrix metalloproteinase 9, angiostatins, SDMA, homoarginine, Fabry disease, Fabry cardiomyopathy, vasculopathy, endothelial dysfunction

## Abstract

**Background:** Fabry disease (FD) is characterized by early development of vasculopathy and endothelial dysfunction. However, it is unclear whether these findings also play a pivotal role in cardiac manifestation. As Fabry cardiomyopathy (FC) is the leading cause of death in FD, we aimed to gather a better insight in pathological mechanisms of the disease.

**Methods:** Serum samples were obtained from 17 healthy controls, 15 FD patients with and 7 without FC. FC was defined by LV wall thickening of >12 mm in cardiac magnetic resonance imaging and serum level of proBNP, high sensitive Troponin T (hsT), and globotriaosylsphingosine (lyso-GB3) were obtained. A multiplex ELISA-Assay for 23 different angiogenesis markers was performed in pooled samples. Markers showing significant differences among groups were further analyzed in single samples using specific Elisa antibody assays. L-homoarginine (hArg), L-arginine, asymmetric (ADMA), and symmetric Dimethylarginine (SDMA) were quantified by liquid chromatography—mass spectrometry.

**Results:** Angiostatin and matrix metalloproteinase 9 (MMP-9) were elevated in FD patients compared to controls independently of the presence of FC (angiostatin: 98 ± 25 vs. 75 ± 15 ng/mL; *p* = 0.001; MMP-9: 8.0 ± 3.4 vs. 5.0 ± 2.4 μg/mL; *p* = 0.002). SDMA concentrations were highest in patients with FC (0.90 ± 0.64 μmol/l) compared to patients without (0.57 ± 0.10 μmol/l; *p* = 0.027) and vs. controls (0.58 ± 0.12 μmol/l; *p* = 0.006) and was positively correlated with indexed LV-mass (*r* = 0.61; *p* = 0.003), hsT (*r* = 0.56, *p* = 0.008), and lyso-Gb3 (*r* = 0.53, *p* = 0.013). Accordingly, the ratio of L-homoarginine to SDMA (hArg/SDMA) was lowest in patients with FC (2.63 ± 1.78) compared to controls (4.16 ± 1.44; *p* = 0.005). For L-arginine, hArg and ADMA no significant differences among groups could be detected, although a trend toward higher ADMA and lower hArg levels could be observed in the FC group. Furthermore, a significant relationship between kidney and cardiac function could be revealed (*p* = 0.045).

**Conclusion:** Elevated MMP-9 and angiostatin levels suggest an increased extracellular matrix turnover in FD patients. Furthermore, endothelial dysfunction may also be involved in FC, as SDMA and hArg/SDMA are altered in these patients.

## Introduction

Fabry disease (FD) is an X-linked recessive multi-systemic storage disorder caused by a decreased activity of the lysosomal enzyme alpha-Galactosidase A (GLA) (1, 2). Due to the deposition of globotriaosylceramide (Gb3) in vascular lysosomes neutral glycosphingolipids accumulate in plasma and various tissues throughout the body (3). Typical manifestations of FD are cardiac, neurological, renal, ocular, dermatological, and gastrointestinal (4–6) with cardiovascular disease as the leading cause of death in FD patients (7). Prognosis of cardiomyopathy is particularly poor (8), thus, appropriate diagnosis and treatment of Fabry associated cardiomyopathy (FC) is crucial. Enzyme Replacement Therapy (ERT) has proven to significantly reduce accumulation of Gb3, especially intracellular deposits in the coronary endothelium (9, 10) and to halt or even partially reverse FC. However, in advanced stages of FD with a severe cardiac phenotype the effectiveness of ERT is profoundly diminished and the disease can even progress (11–13). Therefore, a better understanding of the underlying mechanisms contributing to the development of FC is urgently needed to improve treatment and outcome of FD patients.

Clinical studies provide evidence of increased intima-media thickness (IMT) and impaired artery flow-mediated dilatation in FD (14, 15) indicating an early onset of atherosclerosis in these patients. Moreover, different studies suggest that myocardial fibrosis, detected by cardiac magnetic resonance imaging (cMRI), may contribute to left ventricular remodeling in FD (16, 17). Myocardial fibrosis and subsequent remodeling are caused by an altered extracellular matrix turnover, which is catalyzed by Matrix Metalloproteinases (MMP) including MMP-9 (18). This is in line with the detection of increased serum MMP-9 level in Fabry patients compared to controls (19). Matsunaga et al showed that the inhibition of NO synthase resulted in increased MMP-9 and MMP-2 activities suggesting a link between oxidative stress and extracellular matrix turnover. Several clinical and experimental studies demonstrated inflammatory activity and endothelial nitric oxide synthase (eNOS) alterations in vascular cells of FD patients (20–23). These findings support the hypothesis of early occurring vasculopathy and endothelial dysfunction in FD. Whether these findings play a pivotal role in cardiac manifestation has not yet been sufficiently investigated. Accordingly, the aim of this study was to gather an insight in underlying pathological mechanisms by determining serum markers of endothelial dysfunction, angiogenesis and cardiac function in FD patients with and without FC.

## Methods

### Study population

Serum samples from 15 FD patients with FC, 7 without FC, and 17 healthy controls were collected between September 2014 and December 2016. FD was confirmed by molecular genetic analysis revealing the following mutations: 4x p.N215S, 2x p.E341K, 2x c.1277_1278delAA, 2x c.718_719delAA, p.N320l, p.A143T, p.A230_I232del, p.Q327L, p.A389V, c.717delAA, p.I384N, p.P205T, p.S247P, p.Q327L, p.R227Q. FC was defined by LV wall thickening of >12 mm assessed in cMRI. Furthermore, 11 FD patients with FC and one patient without FC received ERT at the time of blood sampling. The study was in line with the principles outlined in the Declaration of Helsinki and approved by the local ethics committee. All participants gave their written informed consent for participation in the study.

### Enzyme-linked immunosorbent assays

Blood samples were centrifuged at 4000 × G for 10 min at room temperature and obtained serum was aliquoted and stored at −80°C until use. For the multiplex Enzyme-Linked Immunosorbent Assay (ELISA) a Human Angiogenesis Antibody Array Membrane (Abcam PLC, Cambridge, UK, ab169808) was used. Aliquots of serum samples were pooled into three groups: FD with FC (*n* = 15), FD without FC (*n* = 7), and controls (*n* = 17). The assay allowed a simultaneous and semi-quantitative analysis of 23 targets (Angiopoietin 1, Angiopoietin 2, Angiostatin, Endostatin, G-CSF, GM-CSF, I-309, IL-10, IL-1 alpha, IL-1 beta, IL-2, IL-4, I-TAC, MCP-3, MCP-4, MMP-1, MMP-9, PECAM-1, Tie-2, TNF alpha, suPAR, VEGFR2, VEGFR3) and was performed in duplicates. Visualization of membrane signals was performed by ChemiDoc™ MP Imaging System and the densitometry software Image Lab™.

Markers showing significant differences among groups in the multiplex Elisa-Assay were further analyzed in single samples. Specific ELISAs from Abcam were used for MMP-9, angiostatin, soluble urokinase-type plasminogen activator receptor (suPAR), and vascular endothelial growth factor (VEGF) quantification according to the manufacturers' instructions.

### Liquid chromatography—Tandem mass spectrometry (LC-MS/MS) measurements

L-Arginine, L-homoarginine (hArg), asymmetric (ADMA), and symmetric Dimethylarginine (SDMA) were quantified as described previously (24, 25). In brief, 25 μL of EDTA plasma were diluted with 100 μL ^2^H_7_-arginine, ^13^${\text{C}}_{7}^{15}$N_4_-hArg, and ^2^H_7_-ADMA solved in methanol. Proteins were precipitated and residues were derivatised to their butylester derivatives. Twenty microliter of reconstituted samples were injected into the 1200 L Triple Quadrupole MS/MS system (Agilent Technologies, Waldbronn, Germany) chromatography. Analytes were separated on a Polaris C18-Ether column (Agilent Technologies; 50 × 2.0 mm) using an elution gradient of the two mobile phases (A): 1 mL/L formic acid in water and (B) acetonitrile-methanol (50/50, vol/vol) containing 1 mL/L formic acid in water (0:00 min 95/5 (A/B) – 0:30 95/5 – 2:00 50/50 – 2:01 95/5 – 4:00 95/5). The flow rate was 0.3 mL/min. Peak area ratios were calculated with internal standards and external calibration curves prepared in dialysed EDTA plasma. Intra- and interassay coefficients of variation were below 15 % for all analyses.

### Cardiac MRI

Cardiac MRI was performed with a 1.5 Tesla MRI scanner (Achieva, Philips Medical Systems, Philips, Best, The Netherlands). The examination contained a retrospectively gated cine-MRI in cardiac short and long axis orientations using a steady-state free precession (SSFP) sequence to quantify regional and global left ventricular (LV) function and the LV-myocardial mass. Ten minutes after bolus injection of 0.075 mmol/kg Gd-BOPTA (MultiHance®), end-diastolic late gadolinium enhanced (LGE) images were acquired by phase-sensitive inversion recovery (PSIR) sequences to quantify areas of myocardial fibrosis. LGE images were obtained in the LV short-axis as well as in two-, three-, and four-chamber views and quantified using cvi42® software (Circle Cardiovascular Imaging Inc., Calgary, Alberta, Canada). All quantified parameters included LV function, end-diastolic and end-systolic volumes, stroke volume, ejection fraction, left ventricular mass, regional wall thickness, and LGE. Left ventricular mass was indexed to the body surface area calculated by DuBois & DuBois formula.

### Statistical analysis

Obtained data were analyzed using SPSS, version 23. Not normally distributed variables were log transformed if necessary. Assessment of group differences was performed by analysis of variance (ANOVA) or analysis of covariance (ANCOVA) when additionally adjusted for age, sex and eGFR. *Post-hoc* group comparisons were analyzed if the global differences were significant. Correlations were investigated by Pearson's correlation tests. Logistic regression was performed to investigate the relationship between renal and cardiac function. Concentrations are presented as mean ± standard deviation and a *p*-value of < 0.05 was set as statistically significant.

## Results

### Baseline characteristics

Cardiac MRI measurements revealed significantly higher indexed left-ventricular masses of FD patients with FC compared to controls and FD patients without FC as shown in Table 1. Furthermore, similar results could be shown for septal thickness and mean LGE size, which were significantly thicker/higher in FD patients with FC. No differences among groups could be detected for left ventricular ejection fraction and stroke volume. Laboratory values revealed significantly higher levels of proBNP, hsT, and lyso-Gb3 in the cardiomyopathy group. Moreover, patients with FC showed elevated renal function parameters.

**Table 1:** Baseline characteristics, cMRI measurements, and laboratory values.

	**Control (1)**	**Fabry no FC (2)**	**Fabry FC (3)**	***p* (ANOVA)**	***p*** **(1 vs. 2)**	***p*** **(2 vs. 3)**	***p*** **(1 vs. 3)**
*n*	17	7	15				
Age (years)	30.4 ± 6.3	36.3 ± 10.1	47.5 ± 13.0				
Male, *n* (%)	15 (88)	1 (14)	10 (67)				
ERT, *n* (%)	0 (0)	1 (14)	11 (73)				
MSSI	–	5.6 ± 3.2	25.7 ± 7.5				
**cMRI-MEASUREMENTS**							
LVEF (%)	61.1 ± 3.7	70.0 ± 8.8	68.0 ± 14.0	0.224	–	–	–
SV (ml/m^2^)	114.2 ± 20.1	91.4 ± 13.0	100.8 ± 20.0	0.063	–	–	–
LVM indexed to BSA (g/m^2^)	73.1 ± 8.2	73.3 ± 29.9	107.1 ± 34.7	**0.011**	0.988	**0.015**	**0.009**
Septal thickness (mm)	8.9 ± 1.7	8.6 ± 1.1	15.5 ± 3.7	<**0.001**	0.817	<**0.001**	<**0.001**
LGE positive, *n*	0	1	10				
LGE size LV mean (5th *SD*) (%)	0.0	0.014 ± 0.038	4.014 ± 3.970	<**0.001**	0.990	**0.001**	<**0.001**
**LABORATORY VALUES**							
proBNP (pg/ml)	37.7 ± 35.2	67.6 ± 44.3	492.9 ± 634.0	<**0.001**	0.248	**0.012**	<**0.001**
hsT (pg/ml)	6.9 ± 8.8	3.7 ± 0.8	28.8 ± 25.2	**0.001**	0.672	**0.003**	**0.001**
eGFR (ml/min/1.73 m^2^)	104.2 ± 15.8	95.7 ± 19.6	71.9 ± 21.5	<**0.001**	0.321	**0.009**	<**0.001**
Creatinine (mg/dl)	0.94 ± 0.14	0.81 ± 0.14	1.46 ± 1.50	**0.042**	0.347	**0.020**	0.062
ACR (mg/g)	–	30.2 ± 24.2	1534.6 ± 4314.0	–	–	**0.008**	–
Lyso-Gb3 (ng/ml)	–	3.7 ± 3.9	36.0 ± 32.9	–	–	**0.003**	–

*Data are presented as mean ± standard deviation. Significant p-values are marked in bold. Results of the analysis of variance (ANOVA) are shown. If ANOVA was significant post-hoc group comparisons were performed. ACR, albumin-to-creatinine ratio; cMRI, cardiac magnetic resonance imaging; eGFR, estimated glomerular filtration rate using the Chronic Kidney Disease Epidemiology Collaboration (CKD-EPI) formula; ERT, enzyme replacement therapy; Fabry no FC, Fabry patients without Fabry cardiomyopathy; hsT, high sensitive cardiac Troponin T; LGE, late gadolinium enhancement; LGE size LV mean (5th SD), 5th standard deviation of left ventricular mean late gadolinium enhancement size; LVEF, left ventricular ejection fraction; LVM indexed to BSA, left ventricular end-diastolic mass indexed to body surface area; lyso-Gb3, Globotriaosylsphingosine; MSSI, Mainz severity score index; proBNP, prohormone of brain natriuretic peptide; SV, stroke volume*.

### Quantification of angiogenesis markers by enzyme-linked immunosorbent assays

**Figure 1:**
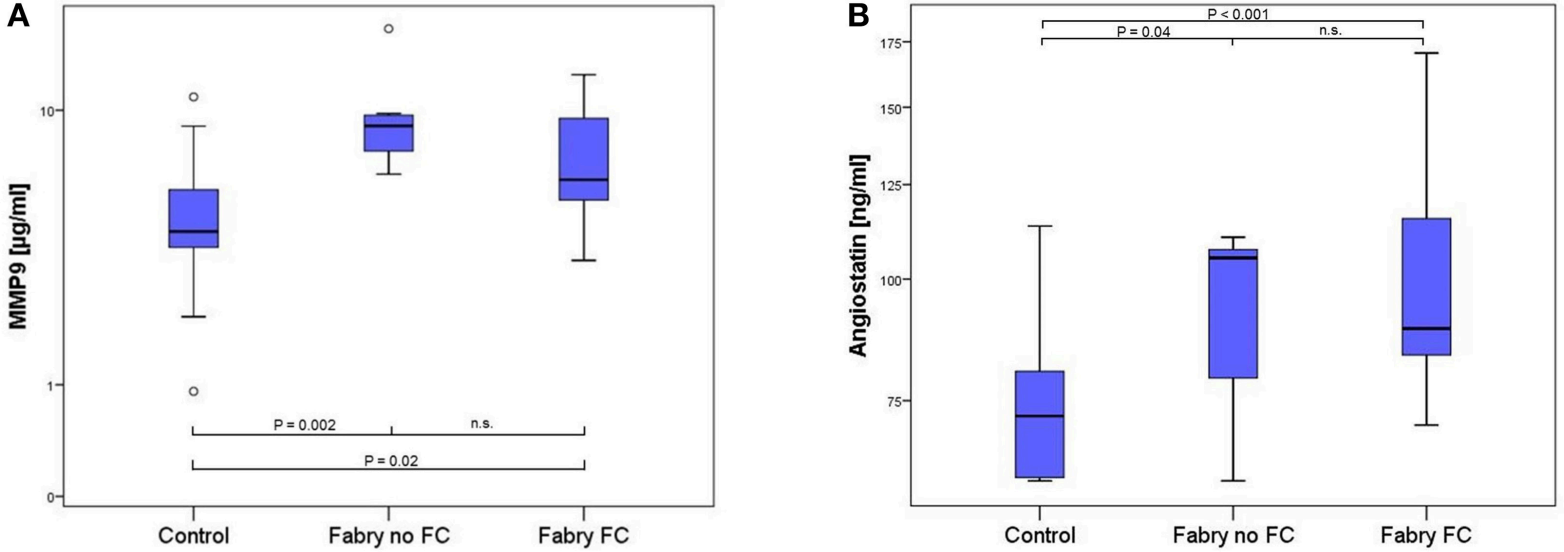
Box-plots of MMP9 **(A)** and angiostatin **(B)** concentration in 17 healthy controls, 7 FD patients without and 15 FD patients with FC. Box plots represent median, 25th and 75th percentiles. Whiskers indicate minimum and maximum without outliers and extremes. On the Y-axis concentrations of the biomarkers are presented (log scale). Brackets indicate *p-*values from the analysis of variance (ANOVA) with *post-hoc* group comparisons. MMP9, matrix metalloproteinase 9; n.s., not significant.

The multiplex ELISA indicated differences among the pooled groups for angiostatin, MMP-9 and suPAR. In Figure 1 the MMP-9 and angiostatin levels from the ensuing ELISA assays are demonstrated. MMP-9 concentrations were 1.51 times higher in FD patients with FC and 2.07 times higher in FD patients without FC compared to controls. The mean MMP-9 level of all FD patients was 1.67 times higher compared to controls (Table 2). Accordingly, angiostatin ELISA provided 1.33 times higher angiostatin levels in FD patients with FC and 1.23 times higher concentrations in FD patients without FC compared to controls. The mean angiostatin level of all FD patients was 1.3 times higher compared to controls (Table 2). However, no significant differences between the two FD groups could be detected for angiostatin and MMP-9. Moreover, neither suPAR nor VEGF concentrations revealed a significant difference between FD patients and healthy controls.

**Table 2:** Markers of endothelial dysfunction.

	**Control (1)**	**Fabry no FC (2)**	**Fabry FC (3)**	***p*** **(ANOVA)**	***p*** **(1 vs. 2)**	***p*** **(2 vs. 3)**	***p*** **(1 vs. 3)**
VEGF (pg/ml)	59.1 ± 51.7	70.1 ± 100.7	89.8 ± 58.6	0.127	–	–	–
suPAR (ng/ml)	1.6 ± 0.5	1.7 ± 0.7	3.2 ± 4.4	0.138	–	–	–
MMP-9 (μg/ml)	5.0 ± 2.4	9.6 ± 3.6	7.3 ± 3.1	**0.004**	**0.002**	0.163	**0.021**
- MMP-9 FD total	5.0 ± 2.4	8.0 ± 3.4^*^		0.002^**^			
Angiostatin (ng/ml)	75.0 ± 15.2	92.9 ± 19.2	101.0 ± 27.3	**0.002**	**0.038**	0.468	**0.001**
- Angiostatin FD total	75.0 ± 15.2	98 ± 25^*^		0.001^**^			
Arginine (μmol/l)	111.4 ± 30.1	114.4 ± 29.6	113.3 ± 31.0	0.944	–	–	–
L-homoarginine (μmol/l)	2.36 ± 0.82	2.24 ± 1.43	1.79 ± 0.79	0.133	–	–	–
ADMA (μmol/l)	0.63 ± 0.13	0.64 ± 0.09	0.77 ± 0.27	0.179	–	–	–
SDMA (μmol/l)	0.58 ± 0.12	0.57 ± 0.10	0.90 ± 0.64	**0.012**	0.959	**0.027**	**0.006**
hArg/ADMA	3.86 ± 1.54	3.48 ± 2.22	2.83 ± 2.14	0.061	–	–	–
hArg/SDMA	4.16 ± 1.44	4.17 ± 3.19	2.63 ± 1.78	**0.017**	0.629	0.078	**0.005**

Data are presented as mean ± standard deviation. Significant p-values are marked in bold. Results of the analysis of variance (ANOVA) are shown. If ANOVA was significant post-hoc group comparisons were performed. ADMA, asymmetric dimethylarginine; FD total, both Fabry disease groups combined; hArg, L-homoarginine; MMP-9, matrix metalloproteinase 9; SDMA, symmetric dimethylarginine; suPAR, soluble urokinase-type plasminogen activator receptor; VEGF, vascular endothelial growth factor.

* Mean ± standard deviation of both FD groups combined (2 + 3)

** *p-value of t-test: 2 + 3 vs. 1*.

### Quantification of endothelial dysfunction markers by LC-MS/MS measurements

**Figure 2:**
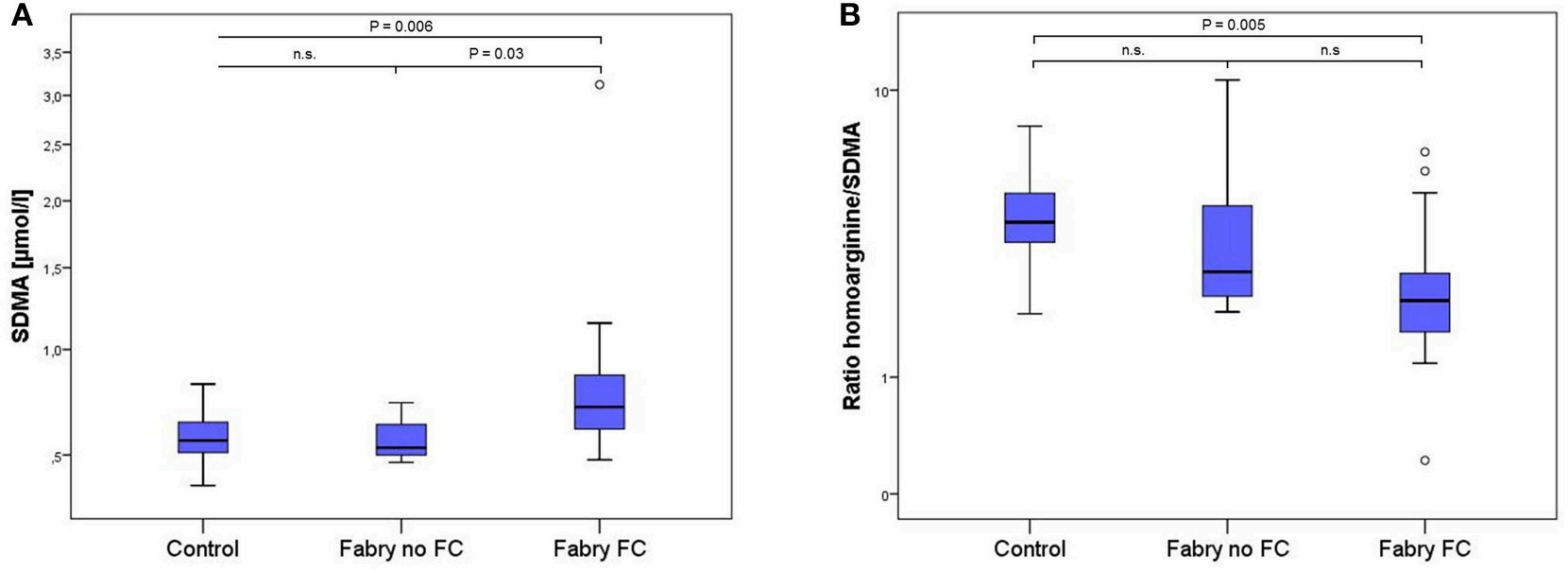
Box-plots of SDMA concentrations **(A)** and the ratio of L-homoarginine and SDMA **(B)** in 17 healthy controls, 7 Fabry patients without and 15 Fabry patients with FC. Box plots represent median, 25th and 75th percentiles. Whiskers indicate minimum and maximum without outliers and extremes. On the Y-axis concentrations of the biomarkers are presented (log scale). Brackets indicate *p*-value from the analysis of variance (ANOVA) with *post-hoc* group comparisons. n.s., not significant; SDMA, symmetric dimethylarginine.

LC-MS/MS measurements revealed 1.4 times higher SDMA concentrations in FD patients with vs. FD patients without FC and equally 1.4 times higher concentrations compared to controls, whereas no statistically significant difference could be shown between FD patients without FC and controls (Table 2, Figure 2). Accordingly, the ratio of L-homoarginine to SDMA (hArg/SDMA) was 0.53 times lower in FD patients with FC compared to controls. For L-arginine, hArg, ADMA and the ratio of hArg/ADMA no significant differences among groups could be detected, although a trend toward higher ADMA concentrations, lower hArg levels and accordingly a lower ratio of hArg/ADMA could be observed in the FD group with existing FC (Table 2).

### Correlations of biomarkers to anthropometric and clinical phenotypes

**Figure 3:**
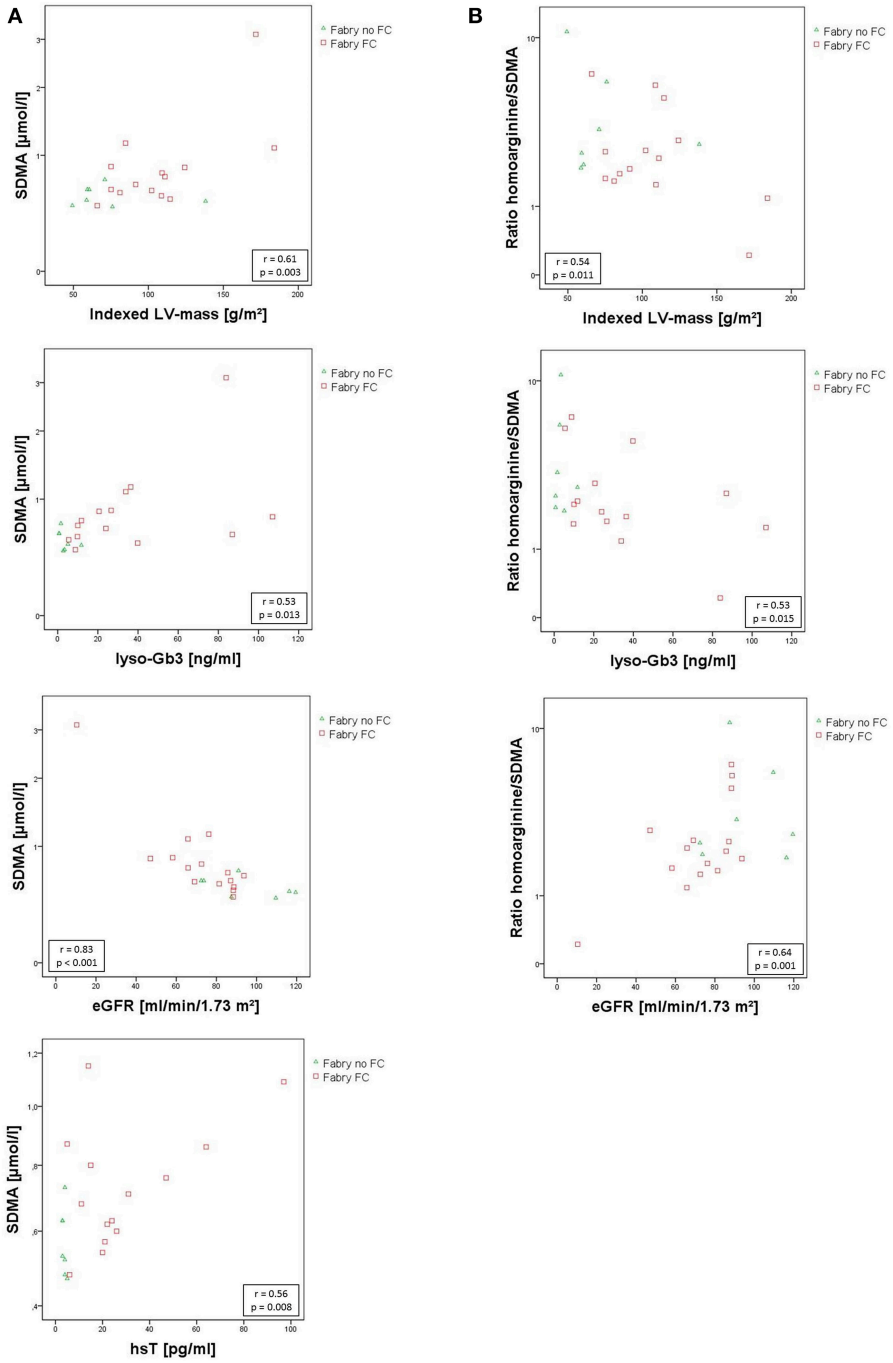
**(A)** Scatterplots of SDMA correlating with indexed left ventricular mass, lyso-Gb3, eGfR, and hsT. **(B)** Scatterplots of hArg/SDMA correlating with indexed left ventricular mass, lyso-Gb3, and eGfR. SDMA concentrations and hArg/SDMA are presented on a log scale. Pearson correlation coefficient *r* and the corresponding *p*-values are given in boxes. eGFR, estimated glomerular filtration rate using the CKD-EPI. Formula; hsT, high sensitive cardiac Troponin T; indexed LV-mass, left ventricular mass indexed to body surface area; lyso-Gb3, Globotriaosylsphingosine; SDMA, symmetric dimethylarginine.

No statistically relevant age or sex dependencies for MMP-9, angiostatin, SDMA, or hArg/SDMA could be observed. As shown in Figure 3A, SDMA was positively correlated with LV-mass, lyso-Gb3, and hsT and negatively correlated with eGFR. Accordingly, as shown in Figure 3B, hArg/SDMA showed a negative correlation to LV-mass, lyso-Gb3, and a positive correlation with eGFR. Moreover, correlations of indexed LV-mass with ADMA, hArg/ADMA, suPAR, lyso-Gb3, cardiac, and renal parameters could be revealed (Table 3). For MMP-9 and angiostatin no significant correlations to any of these variables could be observed (data not shown).

**Table 3:** Correlation to indexed left ventricular mass measured in cMRI.

	***p*-Value**	***r***
SDMA (μmol/l)	0.003	0.61
ADMA (μmol/l)	0.031	0.47
hArg (μmol/l)	0.120	−0.35
hArg/SDMA	0.011	−0.54
hArg/ADMA	0.032	−0.47
suPAR (ng/ml)	0.002	0.64
hsT (pg/ml)	<0.001	0.78
proBNP (pg/ml)	0.002	0.65
lyso-Gb3 (ng/ml)	0.026	0.50
eGFR (ml/min/1.73 m^2^)	0.037	−0.46
Creatinine (mg/dl)	0.014	0.53
ACR (mg/g)	0.018	0.51

*Results from the correlation tests of laboratory values and markers of endothelial dysfunction correlating with indexed left ventricular mass. ACR, albumin-to-creatinine ratio; ADMA, asymmetric dimethylarginine; eGFR, estimated glomerular filtration rate using the Chronic Kidney Disease Epidemiology Collaboration (CKD-EPI) formula; hArg, L-homoarginine; hsT, high sensitive cardiac Troponin T; lyso-Gb3, Globotriaosylsphingosine; proBNP, prohormone of brain natriuretic peptide; r, Pearson's correlation coefficient; SDMA, symmetric dimethylarginine; suPAR, soluble urokinase-type plasminogen activator receptor*.

### Relationship of SDMA and HArg/SDMA to renal function

Analysis of covariance with adjustment for eGFR diminished the significant group differences. Therefore, SDMA and hArg/SDMA are probably dependent on kidney function. Furthermore, an ensuing logistic regression of the eGFR and the two FD groups with and without FC was performed to evaluate the relationship between renal (eGFR) and cardiac function (existence of a FC) in FD patients. This test showed a significant relationship (*p* = 0.045). This finding is supported by the correlation of renal parameters with the LV-mass (Table 3).

## Discussion

The aim of this study was to investigate the role of vasculopathy and endothelial dysfunction in FD with special respect to Fabry associated cardiomyopathy. In blood samples of FD patients generally higher levels of MMP-9 and angiostatin could be detected independently of an existing FC, supporting the hypothesis of an altered extracellular matrix (ECM) turnover in FD.

In patients with FC higher concentrations of SDMA and a decreased ratio of hArg/SDMA could be revealed compared to healthy controls as well as to FD patients without overt cardiomyopathy. These parameters correlate with the ventricular mass as well as with cardiac and renal markers suggesting a potential causal relationship of kidney function and cardiac disease progress.

### MMP-9, angiostatin, and SuPAR

MMP-9 is part of a family of endogenous zinc-dependent endopeptidases. In the myocardium MMPs play an important role for structural integrity of the ECM (18). In patients with familial hypertrophic cardiomyopathy (HCM) an association of MMP-9 with gadolinium enhancement in cardiac MRI was recently described and an important role of the MMP system in cardiac remodeling and fibrosis was proposed (26). In this context, Shah et al. identified significantly higher levels of MMP-9 in 29 FD patients compared to 21 healthy controls and hypothesized that MMP-9 plays an important role in the pathogenesis of FC and might be a valuable surrogate marker for the response to ERT (19). The findings from our study confirm the higher levels of MMP-9 in FD patients. However, a correlation of MMP-9 levels and Fabry associated cardiomyopathy cannot be confirmed.

MMP-9 cleaves matrix-bound plasminogen into angiostatin (27) which is a potent inhibitor of angiogenesis and has been shown to attenuate endothelial cell proliferation and migration (28). In FD the role of angiostatin has not yet been investigated. According to the elevated MMP-9 also Angiostatin concentrations increased in FD compared to controls and showed a trend toward higher concentrations in the FC group compared to FD patients without FC.

Interestingly, a study from Matsunaga et al. could demonstrate that reduced NO production leads to an increase of MMP-2 and MMP-9 activity and higher angiostatin concentrations concluding that NO production influences coronary angiogenesis (29). Furthermore, an experimental study from Takahashi et al. revealed that angiostatin inhibits VEGF-stimulated NO production in human umbilical vein endothelial cells (30). In this context higher MMP-9 and angiostatin levels may contribute to an alteration of NO synthesis in FD. Moreover, an experimental animal study from Givvimani et al. found a switch to higher levels of MMP-9 and anti-angiogenic markers such as angiostatin in the transition from compensatory hypertrophy to decompensated heart failure (31). Both, the effect of MMP-9 and angiostatin on NO synthesis and on a possible transition to decompensated heart failure in FC should be addressed in future studies.

Soluble uPAR is an important regulator of ECM proteolysis and is also involved in MMP activation by plasmin generation (32). For suPAR only a trend toward higher concentrations in FC could be determined, however, a positive correlation with the LV-mass could be found, as shown in Table 3. Soluble uPAR was shown to directly correlate with proteinuria (33). In this context a strong correlation to renal parameters and especially proteinuria (*p* < 0.001, *r* = 0.74) could be revealed.

### Endothelial dysfunction in fabry associated cardiomyopathy

ADMA, SDMA, and hArg are non-proteinogenic amino acids structurally related to L-arginine. hArg has been shown to serve as an alternative substrate for NOS and to inhibit arginase. Thus, it is considered to increase NO formation (34, 35). In addition, low circulating concentrations of hArg have been proposed as a cardiovascular disease risk factor (36). ADMA on the other hand is an endogenous inhibitor of NOS (37), whereas its structural isomer SDMA does not directly interfere with NOS (38). However, SDMA inhibits the tubular L-arginine absorption in kidneys (39) and the *y*^+^ transporter, which mediates the intracellular uptake of L-arginine (40). Therefore, SDMA has an indirect effect on NOS. Both dimethylarginines, ADMA and SDMA, are involved in endothelial dysfunction (41, 42), oxidative stress (43, 44), and atherosclerosis (45). A recent meta-analysis of prospective studies from Schlesinger et al. concluded that both markers are independently associated with cardiovascular disease and all-cause mortality (46).

In this context, the present study reveals a correlation of hArg, ADMA, and the ratio hArg/ADMA with the LV mass. Although group differences of ADMA and hArg showed a trend toward altered concentrations in FC patients, the effect was not significant. Moreover, the ratio of hArg/ADMA was superior compared to the single markers, but it equally missed significance in overall *F*-test of the analysis of variance with a *p*-value of 0.06.

However, in our cohort higher SDMA level in FC patients correlated with LV-mass, hsT, and lyso-Gb3 concentrations indicating the presence of endothelial dysfunction in these patients. Furthermore, this reveals a new possible mechanism of NO alteration in FD as this is the first study demonstrating higher SDMA level in FC patients' sera. Similar results are shown for the hArg/SDMA ratio. Although the alteration of the hArg/SDMA ratio was statistically not superior compared to the higher SDMA concentrations in FC, group differences of this ratio show that hArg might also contribute to a dysfunction of NO synthesis in FC. Due to the antagonistic effects of hArg and SDMA in arginine metabolism a further investigation of this ratio might be reasonable.

The discrepancy between significantly higher SDMA levels without ADMA group differences might be explained by their disparate ways of excretion. SDMA elimination is exclusively renal, whereas ADMA is also enzymatically excreted by the dimethylarginine dimethylaminohydrolases (DDAHs). DDAH-1 for example is highly expressed in the kidney and liver (47) and as FD patients do not typically show liver dysfunction enzymatic ADMA elimination might be sufficient, whereas SDMA accumulates due to the typical early occurring renal insufficiency in FD. In this regard, adjustment for eGFR showed a significant dependency of SDMA and hArg/SDMA to kidney function. Furthermore, an association of eGFR and FC could be revealed. It is well known that diastolic dysfunction caused by impaired LV relaxation may lead to congestive heart failure and consequently to renal insufficiency. In our cohort only 4 patients with FC presented with mild (grade 1) and 1 patient with moderate (grad 2) diastolic dysfunction. Thus, based on these findings one might speculate that renal insufficiency might rather contribute to FC due to an accumulation of SDMA and its negative effect on NO synthesis in the vascular system. However, a causal relationship between renal and cardiac function in Fabry disease has to be proven in further studies.

## Conclusions

This study provides evidence for an altered ECM turnover with higher levels of MMP-9 and angiostatin in FD patients independent of an existing FC. Moreover, patients with FC showed higher SDMA and hArg/SDMA level, which correlated with LV mass, hsT, and lyso-Gb3 concentration but also with impaired renal function. Renal and cardiac function showed a relationship leading to the hypothesis that accumulation of SDMA due to renal insufficiency in FD might contribute to the development of endothelial dysfunction and subsequently lead to FC.

### Limitations

A major limitation of this study is the small sample size of 22 investigated FD patients with only 7 patients without FC compared to more than twice as much patients with FC. Another limitation is the unequal distribution of male and female patients in the FD groups and the fact that this study is a single center study. Moreover, whether the relationship between kidney and cardiac function is causal cannot be proven by this study design.

### Perspectives

To validate these findings multicenter studies including more patients to investigate the effects of MMP-9 and angiostatin on endothelial dysfunction in FD and to clarify the pathological impact of SDMA accumulation in Fabry associated cardiomyopathy are required.

## Ethics statement

This study was carried out in accordance with the principles outlined in the Declaration of Helsinki. The protocol was approved by the local ethics committee of the Ärztekammer Hamburg, Germany (approval no: PV4056).

## Author contributions

JL: writing of the manuscript, experimental work. NL: experimental work, writing assistance. MA: MRI data acquisition, critical review of the data. NM: acquisition of clinical data, critical manuscript review. SL: statistical analysis. KC and ES: supervision and design of experiments, critical review of data analysis and manuscript. MP: experimental design, supervision of data analysis. writing of the manuscript.

### Conflict of interest statement

The authors declare that the research was conducted in the absence of any commercial or financial relationships that could be construed as a potential conflict of interest. The handling Editor declared a shared affiliation, though no other collaboration, with authors JL, NL, MP.

## References

[1] Zarate YA Hopkin RJ. Fabry's disease. Lancet (2008) 372:1427–35. 10.1016/S0140-6736(08)61589-5 18940466

[2] Fabry H. Angiokeratoma corporis diffusum–Fabry disease: historical review from the original description to the introduction of enzyme replacement therapy. Acta Paediatr Suppl. (2002) 91:3–5. 10.1111/j.1651-2227.2002.tb03102.x 12572835

[3] Brady RO Gal AE Bradley RM Martensson E Warshaw AL Laster L. Enzymatic defect in Fabry's disease. Ceramidetrihexosidase deficiency. N Engl J Med. (1967) 276:1163–7. 10.1056/NEJM196705252762101 6023233

[4] Favalli V Disabella E Molinaro M Tagliani M Scarabotto A Serio A. Genetic screening of anderson-fabry disease in probands referred from multispecialty clinics. J Am Coll Cardiol. (2016) 68:1037–50. 10.1016/j.jacc.2016.05.090 27585509

[5] Germain DP Fabry disease. Orphanet J Rare Dis. (2010) 5:30 10.1186/1750-1172-5-30 21092187 PMC3009617

[6] Mehta A Ricci R Widmer U Dehout F Garcia de Lorenzo A Kampmann C. Fabry disease defined: baseline clinical manifestations of 366 patients in the Fabry Outcome Survey. Eur J Clin Invest. (2004) 34:236–42. 10.1111/j.1365-2362.2004.01309.x 15025684

[7] Mehta A Clarke JT Giugliani R Elliott P Linhart A Beck M. Natural course of Fabry disease: changing pattern of causes of death in FOS - Fabry Outcome Survey. J Med Genet. (2009) 46:548–52. 10.1136/jmg.2008.065904 19473999

[8] Felker GM Thompson RE Hare JM Hruban RH Clemetson DE Howard DL. Underlying causes and long-term survival in patients with initially unexplained cardiomyopathy. N Engl J Med. (2000) 342:1077–84. 10.1056/NEJM200004133421502 10760308

[9] Thurberg BL Fallon JT Mitchell R Aretz T Gordon RE O'Callaghan MW. Cardiac microvascular pathology in Fabry disease: evaluation of endomyocardial biopsies before and after enzyme replacement therapy. Circulation (2009) 119:2561–7. 10.1161/CIRCULATIONAHA.108.841494 19414635

[10] Schiffmann R Murray GJ Treco D Daniel P Sellos-Moura M Myers M. Infusion of alpha-galactosidase A reduces tissue globotriaosylceramide storage in patients with Fabry disease. Proc Natl Acad Sci USA. (2000) 97:365–70. 10.1073/pnas.97.1.365 10618424 PMC26669

[11] Weidemann F Niemann M Stork S Breunig F Beer M Sommer C. Long-term outcome of enzyme-replacement therapy in advanced Fabry disease: evidence for disease progression towards serious complications. J Intern Med. (2013) 274:331–41. 10.1111/joim.12077 23586858 PMC4282332

[12] Weidemann F Niemann M Breunig F Herrmann S Beer M Stork S. Long-term effects of enzyme replacement therapy on fabry cardiomyopathy: evidence for a better outcome with early treatment. Circulation (2009) 119:524–9. 10.1161/CIRCULATIONAHA.108.794529 19153271

[13] Pieroni M Camporeale A Della Bona R Sabini A Cosmi D Magnolfi A. Progression of Fabry cardiomyopathy despite enzyme replacement therapy. Circulation (2013) 128:1687–8. 10.1161/CIRCULATIONAHA.113.002799 24100483

[14] Rombach SM van den Bogaard B de Groot E Groener JE Poorthuis BJ Linthorst GE. Vascular aspects of Fabry disease in relation to clinical manifestations and elevations in plasma globotriaosylsphingosine. Hypertension (2012) 60:998–1005. 10.1161/HYPERTENSIONAHA.112.195685 22868390

[15] Kalliokoski RJ Kalliokoski KK Penttinen M Kantola I Leino A Viikari JS. Structural and functional changes in peripheral vasculature of Fabry patients. J Inherit Metab Dis. (2006) 29:660–6. 10.1007/s10545-006-0340-x 16906474

[16] Moon JC Sachdev B Elkington AG McKenna WJ Mehta A Pennell DJ. Gadolinium enhanced cardiovascular magnetic resonance in Anderson-Fabry disease. Evidence for a disease specific abnormality of the myocardial interstitium. Eur Heart J. (2003) 24:2151–5. 10.1016/j.ehj.2003.09.017 14643276

[17] Weidemann F Breunig F Beer M Sandstede J Stork S Voelker W. The variation of morphological and functional cardiac manifestation in Fabry disease: potential implications for the time course of the disease. Eur Heart J. (2005) 26:1221–7. 10.1093/eurheartj/ehi143 15728649

[18] Kelly D Khan SQ Thompson M Cockerill G Ng LL Samani N. Plasma tissue inhibitor of metalloproteinase-1 and matrix metalloproteinase-9: novel indicators of left ventricular remodelling and prognosis after acute myocardial infarction. Eur Heart J. (2008) 29:2116–24. 10.1093/eurheartj/ehn315 18614523 PMC2941717

[19] Shah JS Hughes DA Tayebjee MH MacFadyen RJ Mehta AB Elliott PM. Extracellular matrix turnover and disease severity in Anderson-Fabry disease. J Inherit Metab Dis. (2007) 30:88–95. 10.1007/s10545-006-0360-6 17160618

[20] Shen JS Meng XL Moore DF Quirk JM Shayman JA Schiffmann R. Globotriaosylceramide induces oxidative stress and up-regulates cell adhesion molecule expression in Fabry disease endothelial cells. Mol Genet Metab. (2008) 95:163–8. 10.1016/j.ymgme.2008.06.016 18707907 PMC2593623

[21] Kang JJ Shu L Park JL Shayman JA Bodary PF. Endothelial nitric oxide synthase uncoupling and microvascular dysfunction in the mesentery of mice deficient in alpha-galactosidase A. Am J Physiol Gastrointest Liver Physiol. (2014) 306:G140–6. 10.1152/ajpgi.00185.2013 24232002 PMC3920075

[22] Kaneski CR Moore DF Ries M Zirzow GC Schiffmann R. Myeloperoxidase predicts risk of vasculopathic events in hemizgygous males with Fabry disease. Neurology (2006) 67:2045–7. 10.1212/01.wnl.0000247278.88077.09 17159117 PMC1950664

[23] Shu L Vivekanandan-Giri A Pennathur S Smid BE Aerts JM Hollak CE. Establishing 3-nitrotyrosine as a biomarker for the vasculopathy of Fabry disease. Kidney Int. (2014) 86:58–66. 10.1038/ki.2013.520 24402087 PMC4077934

[24] Atzler D Mieth M Maas R Boger RH Schwedhelm E. Stable isotope dilution assay for liquid chromatography-tandem mass spectrometric determination of L-homoarginine in human plasma. J Chromatogr B Analyt Technol Biomed Life Sci. (2011) 879:2294–8. 10.1016/j.jchromb.2011.06.016 21737361

[25] Schwedhelm E Maas R Tan-Andresen J Schulze F Riederer U Boger RH. High-throughput liquid chromatographic-tandem mass spectrometric determination of arginine and dimethylated arginine derivatives in human and mouse plasma. J Chromatogr B Analyt Technol Biomed Life Sci. (2007) 851:211–9. 10.1016/j.jchromb.2006.11.052 17194630

[26] Roldan V Marin F Gimeno JR Ruiz-Espejo F Gonzalez J Feliu E. Matrix metalloproteinases and tissue remodeling in hypertrophic cardiomyopathy. Am Heart J. (2008) 156:85–91. 10.1016/j.ahj.2008.01.035 18585501

[27] Cornelius LA Nehring LC Harding E Bolanowski M Welgus HG Kobayashi DK. Matrix metalloproteinases generate angiostatin: effects on neovascularization. J Immunol. (1998) 161:6845–52. 9862716

[28] Moser TL Stack MS Asplin I Enghild JJ Hojrup P Everitt L. Angiostatin binds ATP synthase on the surface of human endothelial cells. Proc Natl Acad Sci USA. (1999) 96:2811–6. 10.1073/pnas.96.6.2811 10077593 PMC15851

[29] Matsunaga T Weihrauch DW Moniz MC Tessmer J Warltier DC Chilian WM. Angiostatin inhibits coronary angiogenesis during impaired production of nitric oxide. Circulation (2002) 105:2185–91. 10.1161/01.CIR.0000015856.84385.E9 11994253

[30] Takahashi S Shinya T Sugiyama A. Angiostatin inhibition of vascular endothelial growth factor-stimulated nitric oxide production in endothelial cells. J Pharmacol Sci. (2010) 112:432–7. 10.1254/jphs.10028FP 20308796

[31] Givvimani S Tyagi N Sen U Mishra PK Qipshidze N Munjal C. MMP-2/TIMP-2/TIMP-4 versus MMP-9/TIMP-3 in transition from compensatory hypertrophy and angiogenesis to decompensatory heart failure. Arch Physiol Biochem. (2010) 116:63–72. 10.3109/13813451003652997 20230216 PMC2879167

[32] Smith HW Marshall CJ. Regulation of cell signalling by uPAR. Nat Rev Mol Cell Biol. (2010) 11:23–36. 10.1038/nrm2821 20027185

[33] Sinha A Bajpai J Saini S Bhatia D Gupta A Puraswani M Serum-soluble urokinase receptor levels do not distinguish focal segmental glomerulosclerosis from other causes of nephrotic syndrome in children. Kidney Int. (2014) 85:649–58. 10.1038/ki.2013.546 24429405

[34] Moali C Boucher JL Sari MA Stuehr DJ Mansuy D. Substrate specificity of NO synthases: detailed comparison of L-arginine, homo-L-arginine, their N omega-hydroxy derivatives, and N omega-hydroxynor-L-arginine. Biochemistry (1998) 37:10453–60. 10.1021/bi980742t 9671515

[35] Hrabak A Bajor T Temesi A. Comparison of substrate and inhibitor specificity of arginase and nitric oxide (NO) synthase for arginine analogues and related compounds in murine and rat macrophages. Biochem Biophys Res Commun. (1994) 198:206–12. 10.1006/bbrc.1994.1029 7507318

[36] Atzler D Gore MO Ayers CR Choe CU Boger RH de Lemos JA. Homoarginine and cardiovascular outcome in the population-based Dallas Heart Study. Arterioscler Thromb Vasc Biol. (2014) 34:2501–7. 10.1161/ATVBAHA.114.304398 25189571

[37] Vallance P Leone A Calver A Collier J Moncada S. Accumulation of an endogenous inhibitor of nitric oxide synthesis in chronic renal failure. Lancet (1992) 339:572–5. 10.1016/0140-6736(92)90865-Z 1347093

[38] Bode-Boger SM Scalera F Kielstein JT Martens-Lobenhoffer J Breithardt G Fobker M. Symmetrical dimethylarginine: a new combined parameter for renal function and extent of coronary artery disease. J Am Soc Nephrol. (2006) 17:1128–34. 10.1681/ASN.2005101119 16481412

[39] Tojo A Welch WJ Bremer V Kimoto M Kimura K Omata M. Colocalization of demethylating enzymes and NOS and functional effects of methylarginines in rat kidney. Kidney Int. (1997) 52:1593–601. 10.1038/ki.1997.490 9407505

[40] Closs EI Basha FZ Habermeier A Forstermann U. Interference of L-arginine analogues with L-arginine transport mediated by the y^+^ carrier hCAT-2B. Nitric Oxide (1997) 1:65–73. 10.1006/niox.1996.0106 9701046

[41] Memon L Spasojevic-Kalimanovska V Bogavac-Stanojevic N Kotur-Stevuljevic J Simic-Ogrizovic S Giga V. Assessment of endothelial dysfunction: the role of symmetrical dimethylarginine and proinflammatory markers in chronic kidney disease and renal transplant recipients. Dis Markers (2013) 35:173–80. 10.1155/2013/306908 24167363 PMC3774969

[42] Boger RH. Association of asymmetric dimethylarginine and endothelial dysfunction. Clin Chem Lab Med. (2003) 41:1467–72. 10.1515/CCLM.2003.225 14656027

[43] Schepers E Glorieux G Dhondt A Leybaert L Vanholder R. Role of symmetric dimethylarginine in vascular damage by increasing ROS via store-operated calcium influx in monocytes. Nephrol Dial Transplant. (2009) 24:1429–35. 10.1093/ndt/gfn670 19059932

[44] Sydow K Munzel T. ADMA and oxidative stress. Atheroscler Suppl. (2003) 4:41–51. 10.1016/S1567-5688(03)00033-3 14664902

[45] Miyazaki H Matsuoka H Cooke JP Usui M Ueda S Okuda S. Endogenous nitric oxide synthase inhibitor: a novel marker of atherosclerosis. Circulation (1999) 99:1141–6. 10.1161/01.CIR.99.9.1141 10069780

[46] Schlesinger S Sonntag SR Lieb W Maas R. Asymmetric and symmetric dimethylarginine as risk markers for total mortality and cardiovascular outcomes: a systematic review and meta-analysis of prospective studies. PLoS ONE (2016) 11:e0165811. 10.1371/journal.pone.0165811 27812151 PMC5094762

[47] Tran CT Fox MF Vallance P Leiper JM. Chromosomal localization, gene structure, and expression pattern of DDAH1: comparison with DDAH2 and implications for evolutionary origins. Genomics (2000) 68:101–5. 10.1006/geno.2000.6262 10950934

